# *Schisandra* Extract and Ascorbic Acid Synergistically Enhance Cognition in Mice through Modulation of Mitochondrial Respiration

**DOI:** 10.3390/nu12040897

**Published:** 2020-03-25

**Authors:** Yunseon Jang, Jae Hyeon Lee, Min Joung Lee, Soo Jeong Kim, Xianshu Ju, Jianchen Cui, Jiebo Zhu, Yu Lim Lee, Eunji Namgung, Han Wool John Sung, Hong Won Lee, Min Jeong Ryu, Eungseok Oh, Woosuk Chung, Gi Ryang Kweon, Chun Whan Choi, Jun Young Heo

**Affiliations:** 1 Department of Biochemistry, Chungnam National University School of Medicine, Daejeon 35015, Korea; harhie@naver.com (Y.J.); toematos@naver.com (J.H.L.); rmj1102@hanmail.net (M.J.L.); aaron0506@naver.com (S.J.K.); juxianshu1214@gmail.com (X.J.); neilcuijc@gmail.com (J.C.); zhujiebo2017@163.com (J.Z.); lyl315@naver.com (Y.L.L.); eunji_ng@naver.com (E.N.); johnsungg@gmail.com (H.W.J.S.); lhwon9092@naver.com (H.W.L.); mjryu@cnu.ac.kr (M.J.R.); mitochondria@cnu.ac.kr (G.R.K.); 2 Department of Medical Science, Chungnam National University School of Medicine, Daejeon 35015, Korea; woosuk119@gmail.com; 3 Infection Control Convergence Research Center, Chungnam National University School of Medicine, Daejeon 35015, Korea; 4 Department of Neurology, Chungnam National University Hospital, Daejeon 35015, Korea; doctor_oh@daum.net; 5 Department of Anesthesiology and Pain Medicine, Chungnam National University Hospital, Daejeon 35015, Korea; 6 Department of Anesthesiology and Pain Medicine, Chungnam National University School of Medicine, Daejeon 35015, Korea; 7 Natural Product Research Team, Biocenter, Gyeonggido Business and Science Accelerator, Suwon 16229, Korea

**Keywords:** schisandra extract, ascorbic acid, mitochondria, synaptic plasticity, hippocampus

## Abstract

Cognitive decline is observed in aging and neurodegenerative diseases, including Alzheimer’s disease (AD) and dementia. Intracellular energy produced via mitochondrial respiration is used in the regulation of synaptic plasticity and structure, including dendritic spine length and density, as well as for the release of neurotrophic factors involved in learning and memory. To date, a few synthetic agents for improving mitochondrial function have been developed for overcoming cognitive impairment. However, no natural compounds that modulate synaptic plasticity by directly targeting mitochondria have been developed. Here, we demonstrate that a mixture of *Schisandra chinensis* extract (SCE) and ascorbic acid (AA) improved cognitive function and induced synaptic plasticity-regulating proteins by enhancing mitochondrial respiration. Treatment of embryonic mouse hippocampal mHippoE-14 cells with a 4:1 mixture of SCE and AA increased basal oxygen consumption rate. We found that mice injected with the SCE-AA mixture showed enhanced learning and memory and recognition ability. We further observed that injection of the SCE-AA mixture in mice significantly increased expression of postsynaptic density protein 95 (PSD95), an increase that was correlated with enhanced brain-derived neurotrophic factor (BDNF) expression. These results demonstrate that a mixture of SCE and AA improves mitochondrial function and memory, suggesting that this natural compound mixture could be used to alleviate AD and aging-associated memory decline.

## 1. Introduction

Brain mitochondria effectively supply the adenosine triphosphate (ATP) necessary for maintaining neuronal activity and function, which, in turn, are responsible for learning and memory [1]. Intact and healthy mitochondria are indispensable for synapse formation and dendritic remodeling [2]. Because amyloid β (Aβ) is taken up and subsequently imported into mitochondria, mitochondrial dysfunction is prominent in Alzheimer’s disease (AD) model mice, which show a progressive impairment in cognition [3]. In rat hippocampal slices, blockade of mitochondrial ATP production leads to deficits in long-term potentiation (LTP)—a critical mechanism for learning and memory.

Prevention and treatment of cognitive impairment is the goal of dementia and AD treatment. Natural compounds, such as resveratrol and green tea, are considered AD preventive therapeutics that reduce Aβ40 levels in cerebrospinal fluid and plasma [4] or enhance cognition, as confirmed by a mini-mental state examination (MMSE); however, only a few such agents are in clinical trials [5,6]. FDA-approved drugs, including Aricept (donepezil) and Razadyne (galantamine), both of which are cholinesterase inhibitors, prevent memory deficits, but cause side effects such as nausea, vomiting, and muscle cramps [7]. Other agents include minocycline, a tetracycline antibiotic that targets neuroinflammation in AD [8], and memantine, an N-methyl-D-aspartate (NMDA) receptor antagonist that attenuates damage caused by reactive oxygen species (ROS) in the brain [9]. Although mitochondrial dysfunction is a causative factor in memory deficits in AD [3], how mitochondrial modulation by natural compounds alters the expression of key synaptic proteins is not known. Thus, we explored the effect of mitochondrial-modulating natural compounds on hippocampal learning and memory.

*Schisandra chinensis* extract (SCE) has been reported to exert neuroprotective effects [10], including amelioration of cognitive deficits in a mouse model of chronic, unpredictable mild stress [11], and improvement of synaptic morphology and plasticity in ovariectomized mice [12]; it also protects against oxidative stress in hepatocytes [13]. Supplementation with ascorbic acid (AA), a water-soluble vitamin C found in plants, has been reported to prevent impairment of synaptic plasticity and hippocampal LTP attributable to oxidative damage induced by lead, a neurotoxic metal [14]. High doses of AA have also been shown to reduce amyloid plaque accumulation in AD model mice. However, whether SCE and AA directly regulate mitochondrial activity is still unknown. Mixtures of bioactive natural plant extract or nutrients are known to produce synergistic effects [15,16]. Because SCE and its metabolites can induce ROS in cells [10,17] and AA is known as an antioxidant [18,19], combining SCE with AA would have the benefit of reducing the oxidative stress of ROS which is increased by enhancement of mitochondrial respiration. In the present study, we demonstrated that a mixture of the natural compounds SCE and AA acted through modulation of mitochondria—the main contributors to intracellular ATP production—to synergistically enhance memory storage and recognition memory in association with an increase in the expression of synaptic plasticity-regulating proteins.

## 2. Materials and Methods

### 2.1. Cell Culture 

mHippoE-14 mouse embryonic hippocampal cell line was cultured in Dulbecco’s Modified Eagle’s medium (DMEM, Sigma-Aldrich, MO, USA), 10% FBS (Hyclone, MA, USA), 1% penicillin and streptomycin (Hyclone, MA, USA) at 37 °C under 5% CO_2_ and 21% O_2_ condition. 

### 2.2. Plant Material and General Procedures of Natural Products

The *Schisandra chinensis* was purchased from TCM market in Seoul, Korea in September 2017. Voucher specimens (GL0680) were authenticated by us and were deposited by Dr. Chun Whan Choi at the herbarium of Bio-center, Gyeonggi Institute of Science & Technology Promotion, Suwon, South Korea. ^1^H and ^13^C NMR experiments were performed on a Bruker Ascend 700 MHz spectrometer with tetramethylsilane (TMS). LC-ESI-MS were obtained on a Triple TOF 5600+ instrument (AB SCIEX, MA, USA) and HRESI-MS on a LTQ Orbitrap XL instrument (Thermo Scientific, MA, USA). Thin Layer Chromatography (TLC) was conducted on Silica gel 60 F_254_ (Merck, Darmstadt, Germany) and Silica gel 60 RP-18 F_254S_ (Merck, Darmstadt, Germany) plates. Column chromatography (CC) was performed using Silica gel 60 (70~230 mesh, Merck, Germany), ODS-A (12 nm S-7 μm, YMC GEL, Tokyo, Japan), and preparative high performance liquid chromatography (HPLC) was performed on LC-8A (Shimadzu, Japan).

### 2.3. Isolation and Determination of Schisandrin from Schisandra Chinensis Extract

The dried *Schisandra chinensis* (600 g) were ground and powdered. The powder was extracted with 70% EtOH two times at room temperature (each time for 2 days) and the combined extracts were concentrated under vacuum at 40 °C to yield 75.7 g (92 brix) of extract. The extract (70 g) was separated by Diaion HP-20 chromatography using gradient mixtures as eluents (water: MeOH; 100:0, 70:30, 30:70, 0:100), (F001-004). Compounds 1 (63.2 mg) were isolated from F003 by preparative HPLC (column: YMC-Pack ODS-A, 5 μm, 250 × 20 mm I.D., Japan, 8 mL/min, 10–35% MeCN, 40 min). Structures of Compound **1** (schisandrin) were elucidated by chemical evidence on the basis of NMR spectroscopic and MS data, and as well as by comparison with those reported.

### 2.4. Oxygen Consumption Rate (OCR) Measurement

mHippoE-14 cells were plated 2 × 10^4^ cells at each well and incubated in media containing SCE, ascorbic acid (AA, Sigma-Aldrich, MO, USA) or SCE and AA mixture (10 ug/mL) for 24 h. After measurement of basal OCR, ATPase inhibitor oligomycin A (20 µg/mL, Sigma-Aldrich, MO, USA), uncoupler carbonyl cyanide 3-chlorophenylhydrazone (CCCP, 50 µM, Sigma-Aldrich, MO, USA) and mitochondrial complex I inhibitor rotenone (20 μM, Sigma-Aldrich, MO, USA) were sequentially added to each well and OCR was measured at 37 °C using XF24 analyzer (Seahorse, MA, USA). 

### 2.5. Animal Experiments

Eight-week-old male C57BL/6 mice were used for the experiments. These mice were maintained at 22 °C under a 12-h light-dark cycle. Animal experiments were performed from 9 a.m. to 6 p.m., which is light phage of the cycle. Animal experiments were approved by the Institutional Animal Care and Use Committee of Chungnam National University (Ethical approval number, 201903A-CNU-46). Eight-week-old male mice were intraperitoneally injected with SCE, AA, or a mixture of SCE and AA (10 mg/kg per injection) three times at 24-h intervals. Control (CN) mice were injected with saline and an equal amount of N-methyl-2-pyrrolidone (NMP; Sigma-Aldrich, MO, USA) as the SCE-injection group. Behavioral testing was performed after injection. We used five mice per group for behavior tests. We performed Western blotting three times independently with different samples.

### 2.6. Behavioral Tests

**Open-field test:** Mice injected with saline (CN), SCE, AA, or an SCE and AA mixture were placed in a 40 × 40 × 40 cm box and their movements were recorded for 60 min. Distance moved and heat-map images were obtained using EthoVision XT 11.5 software.

**Fear memory test:** Fear tests consisting of fear conditioning, contextual memory, and cued-fear memory tests were performed using a fear chamber (Coulbourn Instruments, MA, USA). Mice were placed in the fear chamber and fear-conditioning tests were performed as three trials of stimulus, consisting of a 20-s tone (3 kHz, 80 dB), and 1 s of 0.4-mA electric shock after a 5-min habituation period. The duration of each trial was 1 min. Contextual-fear memory was analyzed 24 h after fear conditioning by placing mice in the same chamber for 5 min without a stimulus. Cued-fear memory tests were performed 24 h after contextual-fear memory tests by placing mice in a novel chamber and applying the same tone used in the fear-conditioning test for 3 min after a 5-min habituation period. The threshold for freezing time was set to 0.75 s, and freezing time and testing time, expressed as a percentage, were calculated using FreezeFrame software.

**Novel object-recognition (NOR) test:** The NOR test consisted of two video-recorded sessions. In session 1, two identical objects were placed at an equal distance from the center of the base of a cylinder (diameter 25 cm, height 40 cm). Mice were placed in the cylinder and allowed to freely explore the objects and space for 10 min. After this, mice were returned to their cage and the cylinder was cleaned with 70% ethanol. In session 2, one object was replaced with a novel object with a different texture and shape, and mice were allowed to explore the objects for 10 min. Time spent touching the objects with the forepaw and sniffing was considered as exploration and was measured.

### 2.7. Protein Extraction and Western Blot

Proteins of mice hippocampus and frontal cortex was prepared after 3 injections of SCE, AA, SCE and AA mixture (10 mg/kg) using an RIPA buffer consisting of 50 mM Tris–HCl pH 7.5, 150 mM NaCl, 0.1% SDS, 0.5% deoxycholate and 1% Nonidet P-40. A 10% phosphatase inhibitor and 10% protease inhibitor cocktail (Roche, Basel, Switzerland) was added before using the RIPA buffer. Next, 12 μg of protein was mixed with a 5X sample buffer and loaded on SDS-PAGE gel. The Protein sample was run on gel by electrophoresis and transferred by 200 mA to polyvinylidene fluoride (PVDF) membrane (Millipore, MA, USA). The membrane was blocked by 5% skim milk and incubated with primary antibodies including anti-PSD95 (Thermo Scientific, MA, USA), GluR1 (Abcam, Cambridge, UK), GAD65 (Abcam, Cambridge, UK), Gephyrin (Synaptic Systems, Goettingen, Germany), and α-Tubulin (Sigma-Aldrich, MO, USA) antibodies at 4 °C overnight. The membrane was incubated with a secondary anti-mouse or rabbit IgG horseradish peroxidase antibody (HRP, Pierce Biotechnology, MA, USA), which corresponds to the primary antibody host, for 1 h at room temperature and each protein band was visualized by the ECL system (Thermo Scientific, MA, USA). For ECL detection, we used medical X-ray film blue (AGFA CP-BU NEW, Belgium), developer solution, and fixer solution for the ECL detection.

### 2.8. Statistical Analysis

Statistical analysis of data was performed by Prizm5 software (Graphpad, CA, USA) and data are presented as mean ± SD. One-way ANOVAs and two-way ANOVAs were used for analyzing significance of difference between groups and *p* < 0.05 was considered statistically significant. 

## 3. Results

### 3.1. SCE-AA Mixture Enhances Mitochondrial Respiration in Hippocampal Neurons

**Figure 1:**
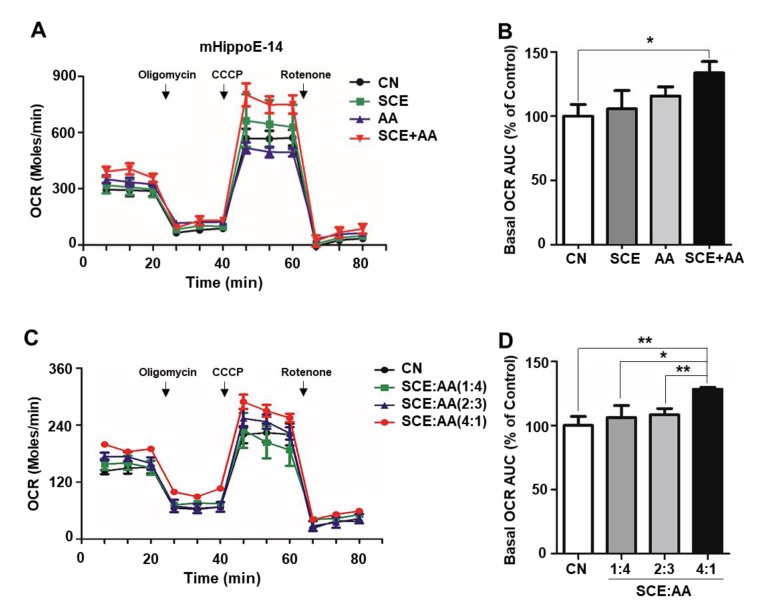
SCE-AA mixture increases oxygen consumption rate (OCR) in hippocampal neuronal cells. (**A**,**B**), OCR of mHippoE-14 cells (2 × 10^4^) incubated in Dulbecco’s Modified Eagle Medium (DMEM) containing Schisandra extract (SCE), ascorbic acid (AA), or an SCE and AA mixture (10 μg/mL) for 24 h was measured with an XF24 analyzer. (**C**,**D**), OCR following treatment with different ratios of SCE and AA was measured in mHippoE-14 cells. Area under the curve of basal OCR was calculated using XF24 software (**B**,**D**). Values are presented as means ± SD of triplicate samples (* *p* < 0.05 vs. corresponding control). CN, control.

Mitochondrial respiration is responsible for supplying the ATP necessary to sustain synapse formation and dendritic remodeling [1]. Because schisandrin C is the predominant compound in SCE, as identified by high-performance liquid chromatography (HPLC) analysis, and has been shown to enhance mitochondrial biogenesis in dental pulp [20], we tested the effects of SCE on mitochondrial respiration in hippocampal neurons (Figure S1). However, we found no change in the mitochondrial oxygen consumption rate (OCR) in response to treatment with SCE alone (10 μg/mL) for 24 h (Figure 1A). It has been reported that combinations of plant metabolites exert synergistic activities, including enhanced antioxidant capacity and anti-inflammatory effects [15,16]. Therefore, we explored whether treatment with a mixture of SCE and AA synergistically altered mitochondrial respiration. As shown in Figure 1A,B, incubation of mHippoE-14 cells derived from the embryonic mouse hippocampus in media containing a mixture of SCE and AA (10 μg/mL, 4:1 ratio) for 24 h enhanced basal OCR, measured using an XF24 analyzer, increasing it by 33.7% compared with controls. Maximum respiration was also increased in the group treated with a mixture of SCE and AA (Figure 1A,B). We next examined the effect of different ratios of SCE and AA on OCR in hippocampal cells. We found that basal OCR increased significantly with increasing ratios of SCE, reaching the highest level at an SCE:AA ratio of 4:1. At mixing ratios of 1:4 and 2:3, the effect of SCE-AA mixtures was comparable to those of controls (Figure 1C,D). The extracellular acidification rate (ECAR) showed a mild increase in groups of administration of the SCE-AA mixture, but it was also increased by treatment with AA only (Figure S2A,B). Furthermore, a different mixing ratio of SCE and AA increased ECAR with no pattern (Figure S2C,D). Unlike 24-h treatment, short-term treatment of mHippoE-14 cells with an SCE-AA mixture exerted effects on OCR and ECAR comparable to those observed in controls (Figure S3A,B). Taken together, these results suggest that the SCE-AA mixture has synergistic effects on mitochondrial respiration that depend on the proportion of SCE in the mixture. 

### 3.2. SCE-AA Mixture Improves Fear Learning and Memory in Mice

**Figure 2:**
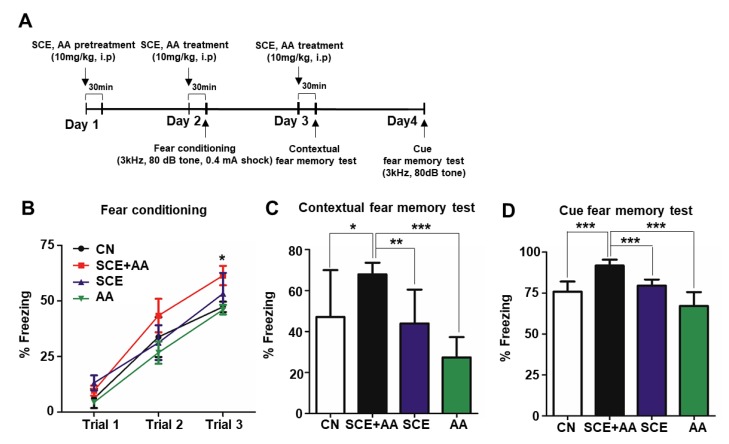
SCE-AA mixture enhances learning and memory. (**A**), Experimental time-line for the fear-memory test. (**B**), Fear-conditioning test in mice treated three times at 24-h intervals with SCE, AA, or an SCE and AA mixture (10 mg/kg). Mice injected with a mixture of SCE and AA showed greater freezing behavior than other groups in trial 3. Freezing time, presented as a percentage of total test time, increased linearly with trial number. (**C**,**D**), Contextual fear memory (**C**) and cued fear memory (**D**) test results, presented as means ± SD (*n* = 5/group; * *p* < 0.05, *** *p* < 0.001 vs. corresponding controls).

Increased mitochondrial respiration is known to produce cognitive enhancement and neuroprotection [3,21]. To verify whether increased mitochondrial respiration in hippocampal neurons induced by an administration of an SCE-AA mixture (Figure 1) affects learning and memory in mice, we performed contextual and cued fear-conditioning tests. Hippocampal plasticity has been implicated in the regulation of fear learning and memory storage [22]. In fear-conditioning tests, we assessed the hippocampal-dependent formation of contextual memory of an electric foot shock in mice by measuring freezing time; in all tests, the threshold for a freezing episode was set at 0.75 s (Figure 2A). In the fear-conditioning period, consisting of a 5-min habituation period and three trials of a 20-s tone and 1-s shock, with each trial separated by 1-min intervals (event 1), mice injected with the SCE-AA mixture spent 61.4% of the total testing time freezing compared with 47.4% for control mice; freezing time in mice injected with SCE or AA only was comparable to that of controls (Figure 2B). In the contextual fear memory test (event 2) performed 24 h after event 1, freezing behavior of mice was measured by tracking movement for 5 min without a stimulus in the same environment as that used for event 1. Subsequent cued fear-memory tests (event 3) were performed using the same 30-min tone as used in event 1. Freezing time, expressed as a percentage of total testing time, for mice in the SCE-AA mixture-injected group was 30.5% higher in event 2 and 17.4% higher in event 3 than in controls (Figure 2C,D). These data support the interpretation that the SCE and AA mixture increases freezing behavior induced not only by contextual fear memory but also cued fear memory, which is mediated by the hippocampus.

### 3.3. Administration of SCE-AA Mixture Enhances Recognition Memory in Mice

**Figure 3:**
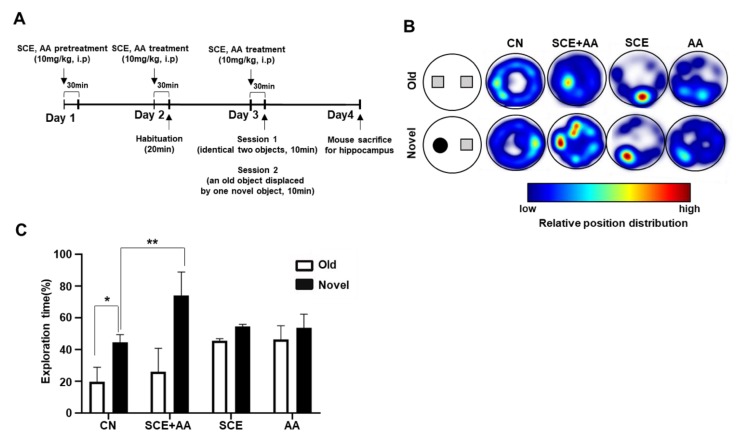
Injection of SCE-AA mixture enhances recognition memory in mice. (**A**), Experimental time-line of the novel object-recognition (NOR) test. (**B**), Heat-map image of the NOR test. (**C**), Exploration time was calculated as a percentage of total test time. Results are presented as means ± SD of three experiments (*n* = 5/group, * *p* < 0.05 vs. corresponding controls).

It has been reported that lesions in the hippocampus or cortex lead to disruptions in object-recognition memory [23,24]. The hippocampus is the region that stores and retrieves long-term recognition memory of an object [25]; thus, object-recognition memory provides an index for assessing hippocampal function. To explore the effect of an SCE-AA mixture on object-recognition memory, we performed novel object-recognition (NOR) tests according to the schedule shown in Figure 3A. The NOR test is divided into two sessions. In session 1, two identical objects were placed equidistant from the center of the base of a cylinder-shaped box, and the time spent by mice in exploring these objects was recorded. In session 2, one of the two objects was replaced by a new object with a different shape and texture, and the test was repeated. Mice injected with a mixture of SCE and AA spent more time in exploration of the novel object compared with controls, as indicated in a heat map (Figure 3B). Specifically, the exploration time for the novel object compared with that for the familiar object increased by an average of 26.6 s in SCE-AA mixture-injected mice and 2.4 s in control mice (Figure 3C). An analysis of exploration times for novel and familiar objects, expressed as a percentage of the total exploration time during session 2 showed a 48.1% greater exploration time for novel objects than familiar objects for mice injected with a mixture of SCE and AA compared with 14.3% for control mice (Figure 3C). Collectively, these results suggest that an SCE-AA mixture significantly enhances hippocampal-dependent object-recognition memory.

### 3.4. An SCE-AA Mixture Induces PSD95 Expression in the Hippocampus

**Figure 4:**
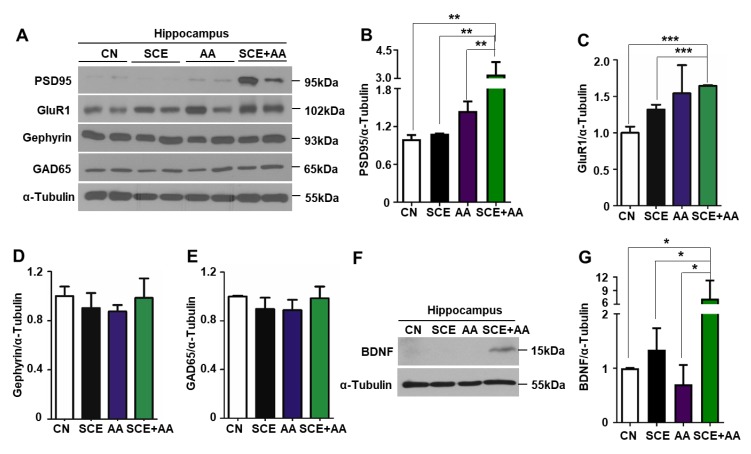
Hippocampal postsynaptic density protein 95 (PSD95) level is increased in mice injected with SCE-AA mixture. (**A**,**F**), C57BL/6 mice were intraperitoneally injected three times with SCE, AA, or an SCE and AA mixture (10 mg/kg). After behavioral tests, mice were sacrificed and total hippocampal protein was extracted and analyzed by Western blotting. (**B**–**G**), PSD95, GluR1, gephyrin, glutamic acid decarboxylase 65-kilodalton isoform (GAD65), and brain-derived neurotrophic factor (BDNF) protein expression levels were measured using ImageJ. Values are presented as means ± SD of triplicate samples (* *p* < 0.05, *** *p* < 0.001 vs. corresponding controls).

**Figure 5:**
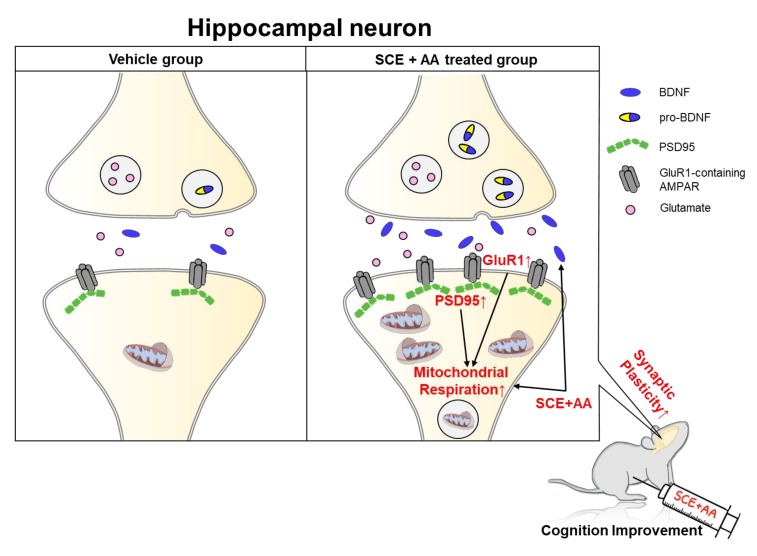
Schematic representation of the effects of an SCE-AA mixture on mitochondrial respiration and synaptic molecule expression in the hippocampus. BDNF, secreted from presynaptic or postsynaptic neurons, astrocytes, or microglia into the synapse, binds to the TrkB receptor and activates signaling cascades responsible for neuronal survival and synaptic plasticity. Our findings suggest that SCE-AA mixture-induced increases in adenosine triphosphate (ATP) production through mitochondrial respiration possibly enhance dendritic spine formation and synaptic plasticity, thereby improving learning and memory storage. The BDNF gene is transcribed in the cytosol in a pro-form and is secreted into the synapse in the activated BDNF form. An SCE-AA mixture induces BDNF expression, leading to reinforcement of synaptic plasticity through increased mitochondrial respiration and upregulation of PSD95.

Learning and memory is regulated by interconnected neural networks and synaptic plasticity. Changes in the synaptic expression of neurotransmitter receptors, such as NMDA and AMPA (alpha-amino-isoxazolepropionic acid) receptors, leads to alterations in plasticity. NMDA receptor NR2 subunits bind the scaffold protein PSD95. AMPA receptors, targets of the neurotransmitter glutamate at excitatory synapses, also associate with PSD95; among the subunits that constitute the AMPA receptor is GluR1. Because the SCE-AA mixture enhanced cognition in mice (Figure 2; Figure 3), we verified whether it also affected synaptic plasticity and/or the expression of excitatory or inhibitory synaptic molecules to regulate learning and memory. To this end, we extracted total protein from the hippocampus of mice injected with the SCE-AA mixture or SCE or AA alone after the NOR test and performed Western blot analyses to determine the synaptic protein expression level. We found a 3.3-fold increase in PSD95 protein level in the hippocampus of mice injected with the SCE-AA mixture compared with controls, as shown in Figure 4A,B. GluR1 expression was increased in the hippocampus of mice injected with either SCE or AA alone, as well as those injected with the mixture of SCE and AA (Figure 4C). In contrast, expression of the inhibitory synaptic proteins gephyrin and GAD65 was comparable in all groups (Figure 4D,E). It has been reported that BDNF, which stimulates neuron development and plasticity [26], increases PSD95 levels in dendritic spines [27]. Therefore, we examined PSD95 protein levels in mice injected with a mixture of SE and AA, or SCE or AA alone. We found that administration of the SCE-AA mixture increased hippocampal BDNF levels by 7.2-fold compared with those in controls, or mice injected with SCE or AA only (Figure 4F,G). Modest increases in PSD95 and BDNF protein expression were observed in the frontal cortex of mice in the SCE-AA mixture-injected group (Figure S4). These results suggest that the SCE-AA mixture improves cognition in mice by stimulating expression of PSD95 protein and thereby regulating excitatory synaptic transmission in the hippocampus, and to a lesser extent in the frontal cortex. The findings further suggest that an increase in PSD95 is associated with BDNF induction and enhanced synaptic plasticity (Figure 5).

## 4. Discussion

Cognitive defects with dendritic loss and impairment of synaptic plasticity are associated with aging and neurodegenerative diseases, including AD [28]. Mitochondrial dysfunction and reduced expression of oxidative phosphorylation (OxPhos) complex proteins are also found in these conditions, resulting in a decrease in the ATP pool. In the present study, we analyzed the beneficial effects of SCE and AA on mitochondrial respiration and behavior. SCE is enriched for a number of bioactive ingredients, including gomisin and schisandrin, as confirmed by HPLC (Figure S1), and is known to enhance cognitive performance and provide neuroprotection [10,13,29]. AA is present in various plants and is used as a dietary supplement. An AA deficiency in the brain impairs cognition and increases amyloid plaque deposition [30]. AA is known to scavenge mitochondria-generated ROS [31], but the effects of SCE or AA on mitochondrial respiration in the brain has not previously been tested. Consistent with previous reports that combinations of plant extracts exhibit augmented biological activity compared with individual plant extracts [32,33], we found that a mixture of SCE and AA at a 4:1 ratio (10 μg/mL) exerted synergistic effects on mitochondrial respiration. However, this effect was absent at lower ratios of SCE in the mixture, implying that not only do SCE and AA in the mixture additively increase mitochondrial activity, the inclusion of AA may contribute to the scavenging of ROS, produced as a result of enhanced mitochondrial respiration. However, which component of SCE is the primary contributor to the efficacy of the SCE-AA mixture remains unknown.

Neurons exploit mitochondrial-derived ATP for the regulation of synaptic plasticity and dendritic remodeling involved in learning and memory function. Hippocampal synaptic plasticity has been implicated in learning and memory, and the loss of mitochondrial ATP production has been reported to impair LTP in the hippocampus [1,2]. Because neurons exclusively depend on glucose metabolisms, deletion of estrogen-related receptor gamma (ERRγ) in the cerebral cortex and hippocampus, which promotes mitochondrial oxidative phosphorylation in the brain, decreases metabolic capacity and impairs LTP and memory formation [34]. Although synthetic agents and chemicals, including vildagliptin and low-dose USP methylene blue are known to increase mitochondrial respiration resulting in amelioration of cognitive impairment in AD [35], there are no FDA-approved plant extracts or natural compounds for improving memory through modulation of synaptic plasticity and mitochondrial activity. We demonstrated that the combination of SCE and AA enhanced mitochondrial respiration of hippocampal neurons and increased expression of key synaptic plasticity-related proteins in the hippocampus upon injection as a mixture (4:1 ratio) in mice.

PSD95 is a major synaptic element that binds to postsynaptic NMDA receptors [36,37]. Glutamatergic neurotransmission mediated by the AMPA receptor is indispensable for synaptic plasticity. In aging and AD model mice, PSD95 expression is diminished, leading to postsynaptic alterations in the cortex and cognitive decline [38,39]. The glutamatergic AMPA receptor is critical for learning and memory and interacts with the PSDs molecule [39]. The GluR1 subunit of the AMPA receptor is downregulated in postmortem brains of AD patients, and LTD decays more rapidly in hippocampal slices from mice with a genetic mutation in GluR1 [40,41]. Unlike PSD95, GluR1 levels in the hippocampus were highest in mice treated with a mixture of SCE and AA, but they were also increased in mice injected with SCE or AA only. We demonstrated that the SCE-AA mixture increased both PSD95 and GluR1—excitatory synaptic transmission regulatory proteins—in the hippocampus to improve cognition in mice through an enhancement of mitochondrial respiration. It has been reported that BDNF induces mitochondrial accumulation at presynaptic sites of hippocampal neurons, and that responses to synaptic stimuli in the form of synaptic plasticity are highly dependent on ATP provided by mitochondria [42,43]. In addition, BDNF treatment increases PSD95 levels and transport to dendritic spines, and enhances synapse formation by postsynaptic neurons [27]. Viewed in this context, our findings suggest that induction of BDNF by the SCE-AA mixture enhances PSD95 protein expression in the hippocampus and frontal cortex, as well as recruitment of mitochondria during learning and memory storage.

Additionally, it is reported that a decrease in synaptic proteins such as synapsin-I, SAP97, and PSD95 were observed in a traumatic brain injury mouse model within 24 h by increasing oxidants and decreasing antioxidants [44]. This implies that short stimulation can cause the change of synaptic protein expression. Furthermore, a decrease in PSD 95 expression also observed at 12 h, 24 h and 72 h after exposure of sevoflurane for 2 h in 2–3 months old mice accompanying impaired short-term memory [45]. From these reports and our results, it is suggestive that the SCE-AA mixture can reduce the expression of PSD95 by short-term treatment. However, the limitation is that we only observed the short-term effects of the SCE-AA mixture and a long-term effect of the SCE-AA mixture in synaptic plasticity needs to be investigated in further studies.

The hippocampus is activated by contextual exposure, and the amygdala reacts to environments recognized as dangerous, whereas the medial prefrontal cortex associates information from these two brain regions and formulates a response [22]. Defects in the hippocampus impair freezing responses to context as well as cued fear conditioning and recognition memory. As we observed in behavioral tests, administration of the SCE-AA mixture enhanced both recognition memory and memory storage (Figure 2 and Figure 3) and was correlated with an induction of PSD95 and BDNF in the hippocampus rather than the frontal cortex (Figure 4). These observations imply that the SCE-AA mixture mainly affects hippocampal memory storage, accompanied by modest effects on frontal cortex-associated memory formation. Whether the combinatorial effects of an SCE-AA mixture on learning and memory observed in healthy mice translates to mitigation of disease-associated cognitive dysfunction in scopolamine-induced memory impairment or AD models will require further investigation.

## 5. Conclusions

Decrease of mitochondrial function is associated with cognitive decline caused by dysregulation of synaptic plasticity. However, there is no clinically used natural compound manipulating mitochondria. Combination of *Schisandra chinensis* extract (SCE) and AA has effects to enhance mitochondrial respiration and to improve cognitive function via induction of a key synaptic protein expression in hippocampus. Our study suggests that SCE and AA mixture could be a therapeutics to prevent cognitive decline.

## Supplementary Materials

The following are available online at https://www.mdpi.com/2072-6643/12/4/897/s1, Materials and Methods: Measurement of oxygen consumption rate (OCR) and extracellular acidification rate (ECAR). mHippoE-14 cells (1 × 10^4^ cells per well) were cultured in media containing SCE (10 ug/mL), ascorbic acid (10 ug/mL, Sigma-Aldrich, MO, USA) or SCE and AA mixture (4:1) for 24 h. Media was discarded and washed with XF media (Agilent, CA, USA) containing 20 mM glucose (VWR Life science, PA, USA). Oligomycin 20 µg/mL (Sigma-Aldrich, MO, USA), CCCP 50 µM (Sigma-Aldrich, MO, USA) and rotenone 20 µM (Sigma-Aldrich, MO, USA) was sequentially added to each well. ECAR was measured by XF24 analyzer (Seahorse, MA, USA) at 37 °C. Figure S1. High-performance liquid chromatography (HPLC) analysis of *Schisandra extract* (SCE). A, HPLC profile of *Schisandra chinensis*. B, HPLC profile of Isolated compound (Schisandrin). C, Structure of Schisandrin. D, UV spectrum of Schisandrin, Figure S2. ECAR of mHippoE-14 cells treated with an SCE-AA mixture for 24 h. A, C, Basal ECAR of mHippoE-14 cells treated with SCE, AA, or an SCE and AA mixture (10 μg/mL per group) for 24 h was measured using an XF24 analyzer. Time points of oligomycin, CCCP and rotenone injection are indicated by arrows. B, D, Area under the curve of basal ECAR was calculated using XF24 software. Values are presented as means ± SD (** *p* < 0.01 vs. control). CN, control. Figure S3. Short-term treatment of mHippoE-14 cells with an SCE-AA mixture does not alter mitochondrial respiration. A, B, OCR (A) and ECAR (B) of mHippoE-14 cells was measured after injection of SCE and AA mixtures with different mixing ratios. Figure S4. BDNF expression is induced by injection of an SCE-AA mixture into the mouse brain. A, B, PSD95 and BDNF protein levels in the frontal cortex were determined by Western blotting. Total protein was extracted from the frontal cortex of CN, SCE, AA, or SCE-AA mixture-injected mice. α-Tubulin was used as an internal control.
