# Supplementary material for: Schisandra Extract and Ascorbic Acid Synergistically Enhance Cognition in Mice through Modulation of Mitochondrial Respiration
Source: Nutrients. 2020 Mar 25;12(4):897. doi: 10.3390/nu12040897 (PMC7230947; doi:10.3390/nu12040897)
Supplement: Supplementary file 1 [file nutrients-12-00897-s001.pdf]

Supplementary Materials

Materials and Methods

Measurement of oxygen consumption rate (OCR) and extracellular acidification rate (ECAR)

mHippoE-14 cells (1x 10^4 cells per well) were cultured in media containing SCE (10ug/ml), ascorbic acid (10ug/ml, Sigma-Aldrich, MO, USA) or SCE and AA mixture (4:1) for 24 hours. Media was discarded and washed with XF media (Agilent, CA, USA) containing 20mM glucose (VWR Life science, PA, USA). Oligomycin 20 μg/ml (Sigma-Aldrich, MO, USA), CCCP 50 μM (Sigma-Aldrich, MO, USA) and rotenone 20 μM (Sigma-Aldrich, MO, USA) was sequentially added to each well. ECAR was measured by XF24 analyzer (Seahorse, MA, USA) at 37 ℃.

Supplementary Figures

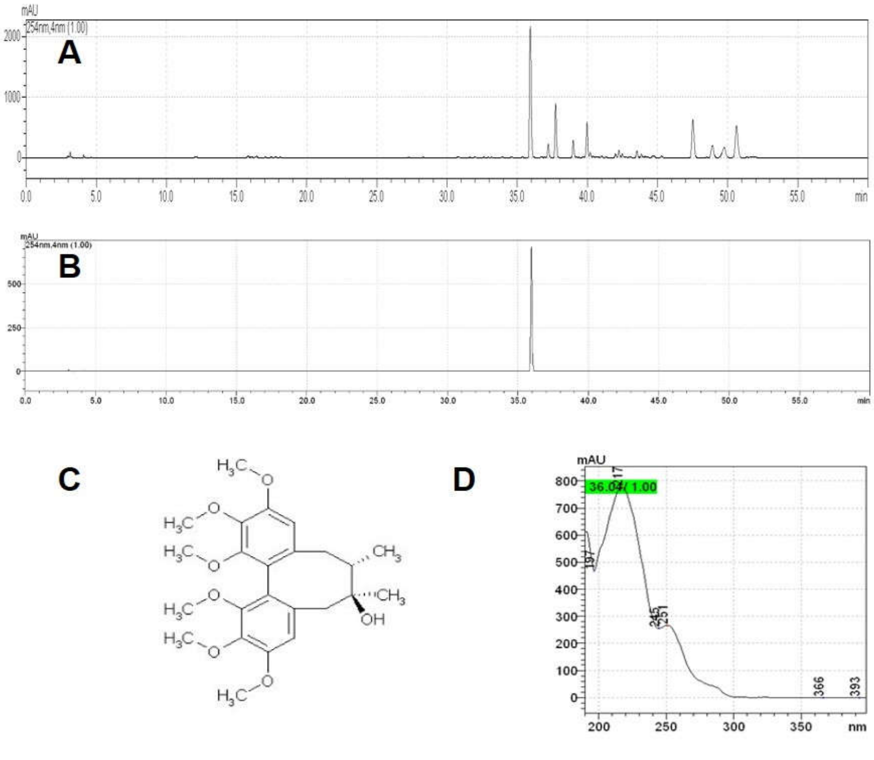

Figure S1. High-performance liquid chromatography (HPLC) analysis of *Schisandra extract* (SCE). A, HPLC profile of *Schisandra chinensis*. B, HPLC profile of Isolated compound (Schisandrin). C, Sturcture of Schisandrin. D, UV spectrum of Schisandrin

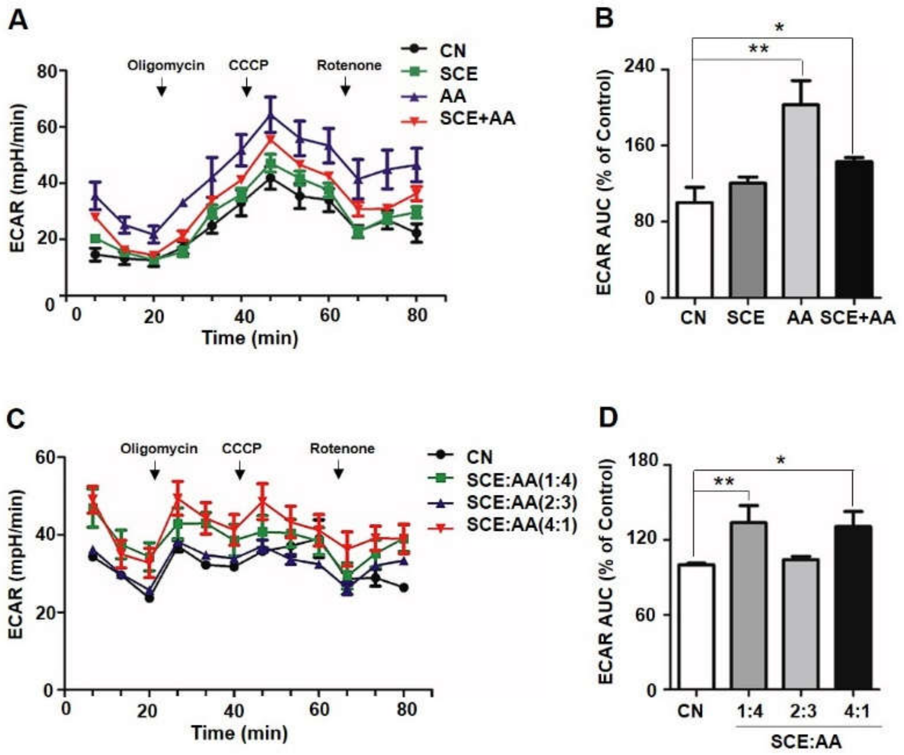

Figure S2. ECAR of mHippoE-14 cells treated with an SCE-AA mixture for 24 hours. A, C, Basal ECAR of mHippoE-14 cells treated with SCE, AA, or an SCE and AA mixture (10 μg/ml per group) for 24 hours was measured using an XF24 analyzer. Time points of oligomycin, CCCP and rotenone injection are indicated by arrows. B, D, Area under the curve of basal ECAR was calculated using XF24 software. Values are presented as means ± SD (**P < 0.01 vs. control). CN, control.

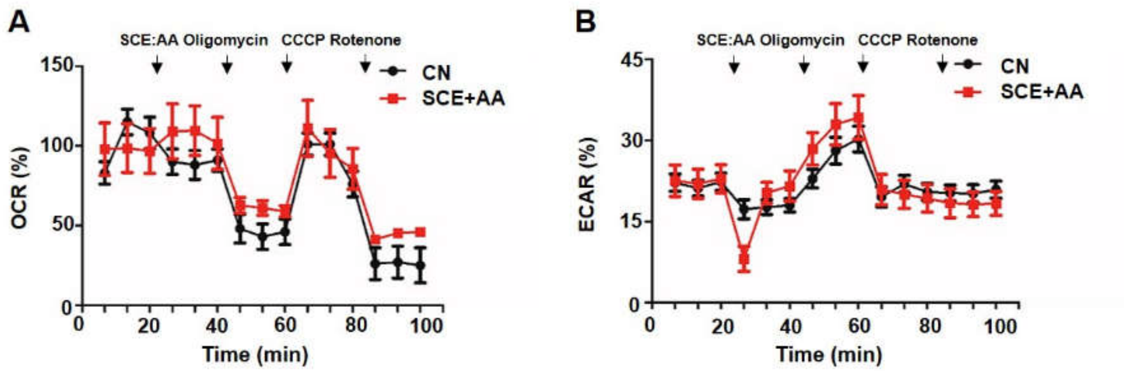

Figure S3. Short-term treatment of mHippoE-14 cells with an SCE-AA mixture does not alter mitochondrial respiration. A, B, OCR (A) and ECAR (B) of mHippoE-14 cells was measured after injection of SCE and AA mixtures with different mixing ratios.

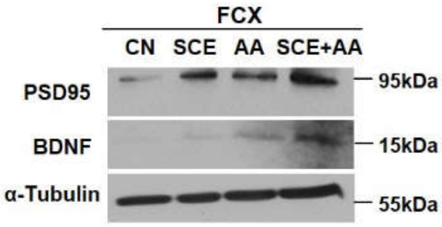

Figure S4. BDNF expression is induced by injection of an SCE-AA mixture into the mouse brain. A, B, PSD95 and BDNF protein levels in the frontal cortex were determined by Western blotting. Total protein was extracted from the frontal cortex of CN, SCE, AA, or SCE-AA mixture-injected mice. *α*-Tubulin was used as an internal control.
